# Halogen bond-driven azo–hydrazone tautomerisation: a computational study

**DOI:** 10.1007/s00894-026-06740-5

**Published:** 2026-05-07

**Authors:** Antti Siiskonen, Arri Priimagi

**Affiliations:** Faculty of Engineering and Natural Sciences, Smart Photonic Materials, Tampere University, Korkeakoulunkatu 3, Tampere, 33720 Finland

**Keywords:** Azobenzene, Halogen bonding, Tautomerisation, Density functional theory

## Abstract

**Context:**

Azo–hydrazone tautomerisation affects the photoswitching behaviour and physical properties of tautomerisable azobenzenes. Non-covalent interactions, such as halogen bonding, can shift the tautomeric equilibrium by stabilising one tautomer over the other. Here, we have used computational methods to study how halogen bonding affects the azo–hydrazone tautomerisation of 2-hydroxy- and 4-hydroxyazobenzenes and their azonaphthalene derivatives. We also studied the effect of alkoxy groups, commonly employed as attachment points when incorporating azobenzenes into functional polymeric systems, on tautomerisation and halogen bonding by systematically replacing ring hydrogens with methoxy groups. In addition, self-complementary halogen-bonded dimers based on 2′-iodo-2-hydroxyazonaphthalene bearing methoxy and nitro groups were studied. Our results show that halogen bonding generally shifts the tautomeric equilibrium towards the hydrazone form. When the azo tautomer is only slightly more stable (∆*G* = 0–2 kcal mol^−1^), halogen bonding can invert the tautomeric preference. External factors such as temperature affect the halogen bonding strength and thereby the tautomeric equilibrium, suggesting that these halogen-bonded systems may offer a tunable platform for sensing applications.

**Methods:**

The Gaussian 16 program was used for geometry optimisations, interaction energy calculations, and to generate the wavefunctions. All calculations were performed using the M06-2X/DGDZVP density functional theory method. The counterpoise correction method by Boys and Bernardi was used to correct for the basis set superposition error. The AIMAll program was used to perform the interacting quantum atoms analyses of the wavefunctions.

**Supplementary information:**

The online version contains supplementary material available at 10.1007/s00894-026-06740-5.

## Introduction

Tautomerism is a type of isomerism wherein two or more isomers exist in equilibrium [1]. A common type of tautomerism is keto–enol tautomerisation, observed in a wide range of chemical compounds [2]. While the term specifically refers to the interconversion between a ketone and its corresponding enol, it is commonly used more broadly to describe processes in which a hydrogen atom shifts position and unsaturated bonds rearrange accordingly. Tautomerism is an important factor to consider, for example, in drug development, as it can affect both pharmacokinetic (e.g. absorption) and pharmacodynamic (e.g. target binding) properties [2, 3]. It can also significantly impact chemical reactivity [4, 5]. External factors, such as solvent polarity, pH, and temperature, can alter the tautomeric equilibrium, thereby changing the relative proportion of each tautomer in a given medium [6]. Since tautomers generally differ in their chemical and physical properties, controlling the equilibrium offers a method for controlling the characteristics of systems containing tautomerisable molecules.

**Fig. 1:**
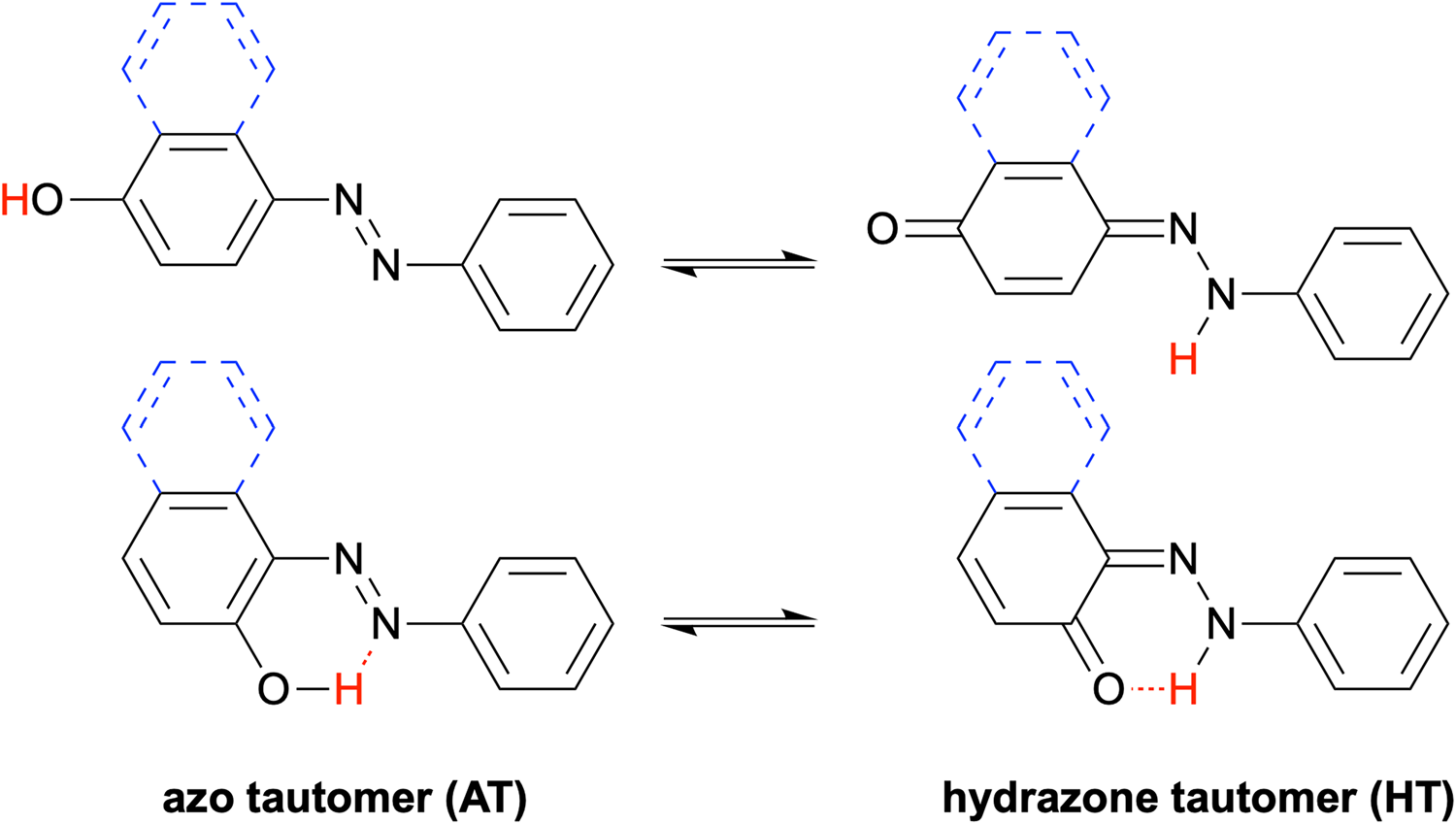
The structures of 4-hydroxyazobenzene (top) and 2-hydroxyazobenzene (bottom) are presented with solid lines. The additional fused aromatic rings in the corresponding hydroxyazonaphthalenes are presented with dashed, blue lines

Azobenzenes have been extensively used as photoswitches due to their cis–trans isomerisation, central to numerous applications [7, 8]. Azobenzenes containing a tautomerisable group (e.g. hydroxyl) in the 2- or 4-position exist in an equilibrium between the azo (AT) and hydrazone (HT) tautomers (Fig. 1). This type of keto–enol tautomerism is referred to as azo–hydrazone tautomerism. Derivatives of 4-hydroxyazobenzene are often employed as photoresponsive molecules in supramolecular chemistry [9–12]. Their azo–hydrazone tautomerism is influenced by water molecules, a property that has been employed in humidity sensing [13, 14]. 2-(4′-Hydroxyazobenzene) benzoic acid is known to bind to the biotin-binding proteins streptavidin and avidin [15–17] and has been studied as a tunable tautomeric switch. The hydrazone form has been identified as the bioactive tautomer, and binding to streptavidin is therefore modulated by the tautomeric equilibrium [18]. 2-Hydroxyazobenzenes have been employed in metal sensing [19, 20], while naphthalene derivatives of hydroxyazobenzenes are present in numerous azo dyes [21]. Understanding their azo–hydrazone tautomerism is important [22], as the hydrazone tautomers of azonaphthalenes often exhibit fluorescence, useful, e.g. for cell imaging [23]. Azobenzene tautomerisation also contributes to the stability and reactivity of the cis- and trans-isomers, thereby influencing the photoswitching performance of azobenzene-based systems [13, 14, 24].

One approach to control tautomerisation is through non-covalent interactions [25–30]. The underlying rationale is that if the less stable tautomer forms stronger interactions, the equilibrium may shift or even invert in its favour. Halogen bonding (XB), which can be rationalised through the σ-hole concept [31, 32], is an excellent choice for this purpose, as it is both directional and tunable [33–37]. Directionality can be exploited, for example, by sterically hindering the XB-accepting site in one tautomer, thereby exerting differences in their bond-forming ability. Tunability, in turn, allows control over the interaction strength and thereby the tautomeric equilibrium. The effect of XB on tautomerisation has been studied experimentally and computationally [26–28, 38, 39]. In β-diketones, a crystallographic study demonstrated an XB-induced shift in tautomeric equilibrium, though the authors stated that “tautomer control through halogen bond still remains elusive” [28]. In 6-halogenated 2-pyridones, which exhibit keto–enol tautomerism, the iodo derivative was found to crystallise as pyridone (keto) instead of the typical pyridinol (enol) form, attributed to XB [38]. Computational studies showed that XB can invert the tautomeric equilibrium of 3-mercapto-1,2-azoles [26] and alter the stability of guanine tautomers [27].

**Fig. 2:**
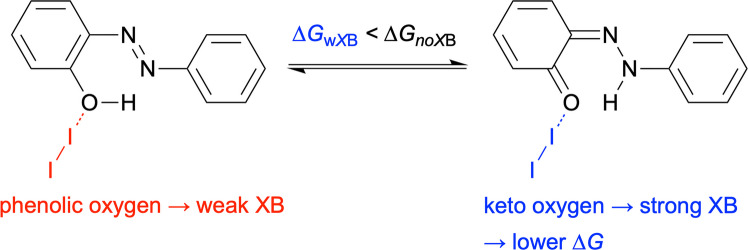
The rationale for halogen bond-driven tautomerisation using 2-hydroxyazobenzene as an example

Herein, we aim to harness halogen bonding to control the tautomerisation of azobenzenes. Using density functional theory (DFT), we computationally investigate halogen-bonded complexes formed between molecular iodine and hydroxyazobenzenes or hydroxynaphthalenes acting as XB acceptors. The focus is on how XB affects the azo–hydrazone equilibrium, governed by the Gibbs free energy difference (∆*G*) between the two tautomers. Phenolic oxygen is a relatively poor XB acceptor as compared to a keto group [40]. Stronger XB to the keto group of the HT tautomer leads to a stronger complex and may therefore lower the ∆*G* value, shifting the equilibrium towards the HT, as illustrated in Fig. 2 for 2-hydroxyazobenzene. To complement the DFT analysis, the interacting quantum atoms (IQA) [41–43] method is used to gain deeper insight into intra- and intermolecular interactions. We further extend the study to self-complementary iodinated azobenzenes capable of acting as both donors and acceptors.

## Methods

The density functional theory method M06-2X/DGDZVP was used for all calculations, since it has been shown to perform well in estimating halogen bond strengths [44]. Geometry optimisations and wavefunction calculations were performed using Gaussian 16, Revision C.02 (Gaussian Inc., Wallingford CT, 2016), with the superfine integration grid applied throughout. The counterpoise correction by Boys and Bernardi [45] was used in all geometry optimisations of the complexes to account for the basis set superposition error (BSSE). The optimised geometries were confirmed to be true minima by frequency analysis, i.e. the absence of imaginary frequencies. AIMAll software [46] was used for the interacting quantum atoms (IQA) analysis and visualisation of the results. Molecular structures for geometry optimisations were constructed using Avogadro [47] (version 1.2.0, avogadro.cc). Chemcraft (build 637 m, https://www.chemcraftprog.com) was used to inspect the results of the geometry optimisations and frequency calculations.

## Results and discussion

**Fig. 3:**
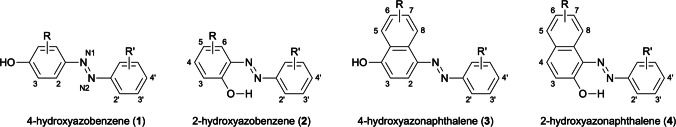
Tautomerisable hydroxyazobenzenes and hydroxyazonaphthalenes used in the study. N1 refers to the azo nitrogen closer to the ring with the hydroxyl group as shown for **1**. R and R′ are either hydrogen or methoxy

In this study, the tautomerisable compounds are methoxy-substituted derivatives of 4-hydroxyazobenzene (**1**) and 2-hydroxyazobenzene (**2**) and their naphthalene analogues (**3** and **4**) (Fig. 3). When azobenzenes are incorporated into polymeric systems, further functionalisation is often achieved via alkoxy groups [48–50]. Therefore, methoxy substituents were introduced to investigate how they affect the tautomeric equilibrium and also the XB interaction. The substitution effect was studied by systematically replacing one of the ring hydrogens with a methoxy group. In addition to exerting inductive and/or mesomeric effects, the substituents may also interact directly with iodine, the azo group, or both. We also studied self-complementary halogen-bonded dimer formation of 2′-iodo-2-hydroxyazonaphthalene (**5**) derivatives.

The core structures (**1**–**4**) have three XB acceptor sites, namely the hydroxyl/keto group (OH/O) and the two azo group nitrogens (N1 and N2). Here, we focus on halogen-bonded complexes where iodine interacts with the OH/O site. In the ATs, the XB interaction is in some cases stronger to N1 or N2 (see Tables S2, S4 and S6), but we are interested in situations where the acceptor site does not change upon tautomerisation, as such large structural changes might, for example, lead to a loss of liquid crystallinity.

For any given compound, the energy difference between the azo and hydrazone tautomers is denoted as ∆*G*. The energy difference between the AT–I_2_ and HT–I_2_ complexes is ∆*G*_wXB_ = ∆*G*(HT−I_2_) - ∆*G*(AT−I_2_), and the change in free energy due to XB is ∆∆*G* = ∆*G *− ∆*G*_wXB_. Therefore, negative ∆∆*G* indicates that XB shifts tautomeric equilibrium towards the HT. The halogen bond strengths in the AT–I_2_ and HT–I_2_ complexes are denoted with ∆*E*_XB–AT_ and ∆*E*_XB–HT_, respectively. The overall effect of substituents on tautomerisation is assessed by comparing free energies, but this does not allow separating the contributions from mesomeric/inductive effects and intramolecular interactions. Nor does it allow contributions from the two XB interactions in bifurcated halogen bonds to be separated.

The IQA method allows calculating interaction energies (*E*_IQA_) between two atoms. The *E*_IQA_ value is the sum of the classical coulombic interaction (*E*_cl_) and the quantum–mechanical exchange–correlation interaction (*E*_XC_). The DFT-calculated halogen bond strengths (∆*E*_XB_) and the IQA interaction energies (*E*_IQA_) cannot be directly compared as *E*_IQA_ describes only the interaction between two atoms but does not consider their interactions with the rest of the molecule. The *E*_IQA_ values can nevertheless be useful in studying specific interactions in bifurcated halogen bonds and for determining whether an interaction is stabilising (*E*_IQA_ < 0) or destabilising (*E*_IQA_ > 0). The *E*_XC_ component is always stabilising (*E*_XC_ < 0), and the overall nature of the interaction therefore depends on *E*_cl_. In other words, even seemingly destabilising interactions can be stabilising if the magnitude of the destabilising Coulombic component is smaller than that of the stabilising exchange–correlation component.

### 4-Hydroxyazobenzene derivatives

**Fig. 4:**
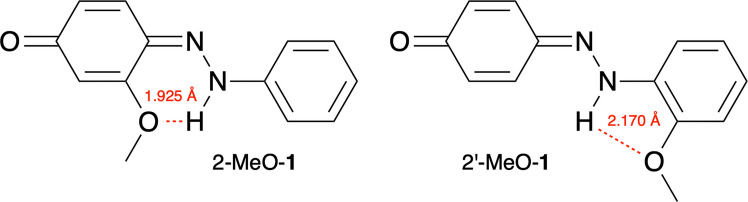
Intramolecular hydrogen bonding in 2-MeO-**1** and 2′-MeO-**1**

For the unsubstituted 4-hydroxyazobenzene (**1**), the energy difference between the AT and HT tautomers is large (∆*G* = 9.29 kcal mol^−1^), and it is strongly favoured as the azo tautomer (AT-**1**). The ∆*G*, ∆*G*_wXB_, and ∆∆*G* values for the derivatives of **1** are presented in Table 1. *Ortho*-methoxy substitution on either ring allows intramolecular hydrogen bonding in the HT, lowering its energy (Fig. 4). The interaction is stronger in 2-MeO-**1** (*E*_IQA_ = −89.48 kcal mol^−1^) than in 2′-MeO-**1** (*E*_IQA_ = −73.69 kcal mol^−1^). Consequently, the shift towards the HT is more noticeable in 2-MeO-**1** (∆*G* = 1.46 kcal mol^−1^) than in 2′-MeO-**1** (∆*G* = 5.28 kcal mol^−1^). Methoxy substituents at other positions do not significantly affect tautomerisation. By combining 2-MeO and 2′-MeO substituents, the equilibrium is shifted further towards the HT, and the tautomeric equilibrium is inverted for 2-MeO-2′-MeO-**1** (∆*G* = −1.35 kcal mol^−1^).

**Table 1:** Halogen bond strengths in the AT–I_2_ (∆*E*_XB–AT_) and HT–I_2_ (∆*E*_XB–HT_) complexes, and the ∆*G*, ∆*G*_wXB_, and ∆∆*G* values for the derivatives of **1**. All values are in kcal mol^−1^

Compound	∆*E*_XB–AT_	∆*E*_XB–HT_	∆*G*	∆*G*_wXB_	∆∆*G*
**1**	−4.33	−7.28	9.29	7.49	−1.80
2-MeO-**1**	−4.33	−8.56	1.46	−0.51	−1.97
3-MeO-**1**	−4.68	−7.96	10.75	8.29	−2.46
2′-MeO-**1**	−4.47	−7.60	5.28	3.45	−1.83
3′-MeO-**1**	−4.32	−7.24	8.94	6.62	−2.32
4′-MeO-**1**	−4.45	−7.52	10.80	8.47	−2.33
2-MeO-2′-MeO-**1**	−4.74	−8.90	−1.35	−4.04	−2.69

The ∆*E*_XB–AT_ and ∆*E*_XB–HT_ values for the derivatives of **1** are presented in Table 1. Iodine binds more strongly to the HTs (*E*_XB–HT_ = −8.90 − –7.24 kcal mol^−1^) than to the ATs (*E*_XB–AT_ = −4.74 − –4.32 kcal mol^−1^). Therefore, halogen bonding shifts the equilibrium towards the HT (∆∆*G* = −2.69 − –1.80 kcal mol^−1^). However, as ∆*G* is high for most monosubstituted compounds, XB does not significantly alter the tautomeric equilibrium and the AT–I_2_ complex is strongly favoured (∆*G*_wXB_ = 3.45 – 8.47 kcal mol^−1^). The only exception is 2-MeO-**1**, for which halogen bonding reverses the tautomeric preference from azo to hydrazone (∆*G*_wXB_ is −0.51 kcal mol^−1^).

### 2-Hydroxyazobenzene derivatives

Despite structural similarities, 2-hydroxyazobenzenes behave somewhat differently from 4-hydroxyazobenzenes (Table 2). The energy difference between the tautomers is lower for **2** (∆*G* = 5.90 kcal mol^−1^) than for **1** (∆*G* = 9.20 kcal mol^−1^), but methoxy substituents overall have a smaller effect on the tautomeric equilibrium of **2** (∆*G* = 3.05 – 6.82 kcal mol^−1^) than on that of **1** (∆*G* = 1.46 – 11.09 kcal mol^−1^). The largest effect on ∆*G* is observed for 4-MeO and 6-MeO substituents, which lower it by 1.87 and 2.85 kcal mol^−1^, respectively. Introducing both substituents causes a notable shift towards the HT, as seen in 4-MeO-6-MeO-**2** (∆*G* = 1.59 kcal mol^−1^).

**Table 2:** Halogen bond strengths in the AT–I_2_ (∆*E*_XB–AT_) and HT–I_2_ (∆*E*_XB–HT_) complexes, and the ∆*G*, ∆*G*_wXB_, and ∆∆*G* values for the derivatives of **2**. All values are in kcal mol^−1^

Compound	∆*E*_XB−AT_	∆*E*_XB–HT_	∆*G*	∆*G*_wXB_	∆∆*G*
**2**	−5.09	−7.67	5.90	4.21	−1.69
3-MeO-**2**	−7.04	−8.59	4.83	3.85	−0.98
4-MeO-**2**	−5.34	−8.40	4.03	1.16	−2.87
5-MeO-**2**	−5.44	−7.82	4.47	2.75	−1.72
6-MeO-**2**	−5.22	−7.95	3.05	2.10	−0.95
2′-MeO-**2**	−6.09	−8.84	4.93	3.07	−1.86
3′-MeO-**2**	−5.07	−7.66	5.79	4.04	−1.75
4′-MeO-**2**	−5.30	−8.17	6.82	4.81	−2.01
4-MeO-6-MeO-**2**	−5.50	−8.69	1.59	−0.47	−2.06

**Fig. 5:**
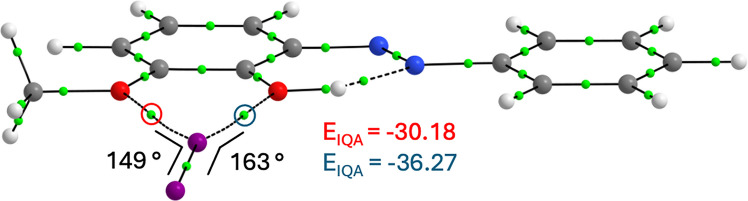
The geometry-optimised structure of the AT-3-MeO-**2**–I_2_ complex. The IQA energy values are in kcal mol^−1^

The XB strength in the AT–I_2_ complexes is approximately −5 kcal mol^−1^, except for 3-MeO-**2** (∆*E*_XB–AT_ =  −7.04 kcal mol^−1^) and 2′-MeO-**2** (∆*E*_XB–AT_ =  −6.09 kcal mol^−1^), as shown in Table 2. In 3-MeO-**2**, bifurcated XB to OH and 3-MeO is observed (Fig. 5), increasing the XB strength by 1.95 kcal mol^−1^ compared to AT-**2**. The interaction with the hydroxyl group (*E*_IQA_ =  −36.27 kcal mol^−1^) is slightly stronger than with 3-MeO (*E*_IQA_ =  −30.18 kcal mol^−1^). As both halogen bonds deviate from the preferred linear geometry, the interactions are weaker than in the AT-**2**–I_2_ complex (*E*_IQA_ =  −39.03 kcal mol^−1^), in which the halogen bond is linear. Despite the sp^3^-hybridised oxygens, AT-3-MeO-**2** forms a planar complex with *I*_2_, presumably to maximise halogen bonding with both oxygens. The HT of 3-MeO-**2** also shows bifurcated XB, but the increase in XB strength due to the 3-MeO substituent is only 0.95 kcal mol^−1^, as the halogen bond angle to 3-MeO is even smaller (141°) than in the AT, resulting in a weaker interaction (*E*_IQA_ =  −28.11 kcal mol^−1^). The only compound for which halogen bonding inverts the equilibrium is 4-MeO-6-MeO-**2** (∆*G*_wXB_ =  −0.47 kcal mol^−1^).

### 4-Hydroxyazonaphthalenes

The additional fused ring in azonaphthalenes significantly stabilises the hydrazone tautomer. Although AT-**3** is more stable than HT-**3**, the energy difference is much smaller (∆*G* = 1.76 kcal mol^−1^) when compared to **1** (∆*G* = 9.29 kcal mol^−1^). The ∆*G*, ∆*G*_wXB_, and ∆∆*G* values for the derivatives of **3** are presented in Table 3. Methoxy substituents have a large effect on the tautomeric equilibrium and, depending on their position, they can significantly stabilise either tautomer. For 2-MeO-**3**, the HT is more stable than the AT (∆*G* =  −9.27 kcal mol^−1^), whereas 5-MeO-**3** shows the opposite behaviour (∆*G* = 8.10 kcal mol^−1^). Similarly to 2-MeO-**1**, the HT of 2-MeO-**3** is stabilised by intramolecular hydrogen bonding that is not present in the AT. In contrast, in 5-MeO-**3**, the AT is stabilised by intramolecular hydrogen bonding between OH and 5-MeO (∆*E*_IQA_ = −129.27 kcal mol^−1^) that is not present in the HT. Also, the HT of 5-MeO-**3** is destabilised by a repulsive interaction between O and 5-MeO (*E*_IQA_ = 139.06 kcal mol^−1^). Intramolecular hydrogen bonding also lowers the energy of HT-2′-MeO-**3** (∆*G* =  −2.87 kcal mol^−1^), but methoxy substituents in other positions have a relatively small effect on the equilibrium.

**Table 3:** Halogen bond strengths in the AT–I_2_ (∆*E*_XB–AT_) and HT–I_2_ (∆*E*_XB–HT_) complexes, and the ∆*G*, ∆*G*_wXB_, and ∆∆*G* values for the derivatives of **3**. All values are in kcal mol^−1^

Compound	∆*E*_XB–AT_	∆*E*_XB–HT_	∆*G*	∆*G*_wXB_	∆∆*G*
**3**	−4.29	−6.86	1.76	−1.27	−3.03
2-MeO-**3**	−4.30	−8.16	−9.27	−12.57	−3.30
3-MeO-**3**	−4.65	−7.61	2.26	−1.25	−3.51
5-MeO-**3**	−5.02	−8.38	8.10	5.95	−2.15
6-MeO-**3**	−4.90	−6.91	1.16	−0.40	−1.56
7-MeO-**3**	−4.31	−7.11	1.20	−1.18	−2.38
8-MeO-**3**	−4.39	−6.12	0.06	−3.33	−3.39
2′-MeO-**3**	−4.41	−7.10	−2.87	−5.11	−2.24
3′-MeO-**3**	−4.28	−6.83	1.52	−1.33	−2.85
4′-MeO-**3**	−4.39	−7.10	2.65	0.97	−1.68

The XB strengths are quite similar for all ATs (*E*_XB–AT_ =  −5.02 – −4.28 kcal mol^−1^) but show some variation in the HTs (∆*E*_XB–HT_ =  −8.38 – −6.12 kcal mol^−1^) as shown in Table 3. However, stronger XB in the HT does not necessarily lead to lower ∆∆*G*. For example, the stabilising OH⋅⋅⋅MeO interaction in AT-5-MeO-**3** strengthens upon halogen bonding (∆*E*_IQA_ =  −3.70 kcal mol^−1^), lowering the energy of the AT–I_2_ complex. Therefore, although the difference in the XB strengths between the tautomers of 5-MeO-**3** is −3.36 kcal mol^−1^, ∆∆*G* is only −2.15 kcal mol^−1^. Interestingly, for several derivatives, halogen bonding ultimately inverts the tautomeric preference from azo to hydrazone.

### 2-Hydroxyazonaphthalenes

The derivatives of **4** behave somewhat differently from those of **1**–**3,** since for most derivatives the HT is more stable than the AT. The ∆*G*, ∆*G*_wXB_, and ∆∆*G* values for the derivatives of **4** are presented in Table 4. Substitution has only a small effect on tautomerisation, but interestingly, depending on the position of the substituent, either the AT or HT is slightly more favoured (∆*G* =  −1.95 – 0.97 kcal mol^−1^). Therefore, even a small change in ∆*G* can invert the tautomeric equilibrium. Some derivatives of **3** also have similar, slightly positive ∆*G* values. However, tautomerisation of **3** requires intermolecular proton transfer, whereas in **4** proton transfer can occur intramolecularly, which likely enables faster tautomerisation. Compound **4** exists as an approximately 1:1 mixture of ATs and HTs (∆*G* =  −0.06 kcal mol^−1^). The largest shift towards HT is observed for 4-MeO-**4** (∆*G* =  −1.95 kcal mol^−1^), whereas 4′-MeO-**4** (∆*G* = 0.97 kcal mol^−1^) shows the largest shift towards AT.

**Table 4:** Halogen bond strengths in the AT–I_2_ (∆*E*_XB–AT_) and HT–I_2_ (∆*E*_XB–HT_) complexes, and the ∆*G*, ∆*G*_wXB_, and ∆∆*G* values for the derivatives of **4**. All values are in kcal mol^−1^

Compound	∆*E*_XB–AT_	∆*E*_XB–HT_	∆*G*	∆*G*_wXB_	∆∆*G*
**4**	−4.86	−6.95	−0.06	−1.81	−1.75
3-MeO-**4**	−6.79	−8.04	−1.05	−1.75	−0.70
4-MeO-**4**	−5.28	−7.85	−1.95	−3.82	−1.87
5-MeO-**4**	−4.95	−7.20	0.30	−1.23	−1.53
6-MeO-**4**	−5.03	−7.06	0.18	−1.04	−1.22
7-MeO-**4**	−4.96	−7.13	0.43	−1.21	−1.64
8-MeO-**4**	−5.05	−7.15	−1.84	−2.77	−0.93
2′-MeO-**4**	−5.90	−7.92	−1.00	−1.71	−0.71
3′-MeO-**4**	−4.85	−6.92	−0.18	−1.11	−0.93
4′-MeO-**4**	−5.08	−7.33	0.97	−0.49	−1.46

Substitution does not noticeably affect the XB strengths of either tautomer, apart from 3-MeO-**4**, which shows the strongest interaction for both tautomers due to a bifurcated halogen bond similar to that observed in 3-MeO-**2** (Fig. 5). As shown in Table 4, halogen bonding does not have a very large effect on tautomerisation (∆∆*G* =  −1.87– −0.70 kcal mol^−1^). However, the ∆*G* values are close to zero in many cases, and even a modest effect suffices to shift the equilibrium from azo to hydrazone. Overall, the derivatives of **4** are interesting for further development, as the ∆*G* values are only slightly positive and the derivatives can tautomerise via intramolecular proton transfer.

### Self-complementary halogen-bonded dimers

**Fig. 6:**
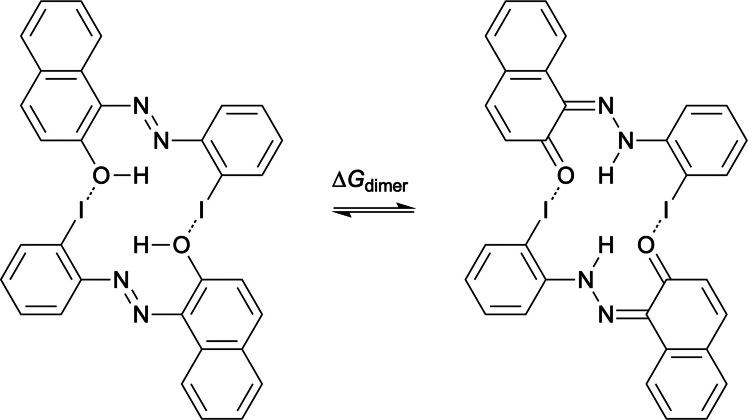
Self-complementary halogen-bonded dimer formation of 2′-iodo-2-hydroxyazonaphthalene (**5**)

We reasoned that iodine in the 2′-position of **4** would be correctly oriented to allow formation of a self-complementary halogen-bonded dimer, as shown in Fig. 6. Compounds **1**–**4** act as XB acceptors, and the electron-donating methoxy groups increase the XB strength by increasing electron density on the acceptor site. Compound **5** acts both as an XB donor and acceptor. In addition to methoxy groups, electron-withdrawing nitro substituents were introduced, as they can increase XB strength by decreasing electron density on the XB donor atom (iodine). The effect of XB on tautomerisation was studied by comparing the tautomeric equilibrium of the monomer (∆*G*) to that in the dimer (∆*G*_dimer_), where ∆∆*G* = ∆*G*_dimer_ − ∆*G*. Therefore, negative ∆∆*G* indicates that dimerisation shifts the equilibrium towards the HT.

The ∆*G*, ∆*G*_dimer_, and ∆∆*G* values for the derivatives of **5** are presented in Table 5. The 2′-iodo substituent has only a small effect on the tautomeric equilibrium (∆*G* = 0.25 kcal mol^−1^ for **5** vs −0.06 kcal mol^−1^ for **4**). Methoxy and nitro substituents also have only moderate effects (∆*G* = −1.52 – 1.33 kcal mol^−1^), but depending on their position, the equilibrium can be shifted towards either the AT or HT. Substitution in the 4- and 4′-positions provides a simple way to control the equilibrium. In 4-MeO-**5,** the equilibrium is shifted towards HT (∆*G* = −1.52 kcal mol^−1^), whereas in 4′-MeO-**5**, it shifts towards AT (∆*G* = 1.33 kcal mol^−1^). Nitro substituents show the opposite trend, and AT is favoured in 4-NO_2_-**5** (∆*G* = 1.13 kcal mol^−1^) and HT in 4′-NO_2_-**5** (∆*G* = −0.96 kcal mol^−1^). When combining the effect of both the methoxy and nitro substituents, the equilibrium can be shifted even further towards the AT or HT. The largest shift towards the AT is observed for 4-MeO-4′-NO_2_-**5** (∆*G* = −2.46 kcal mol^−1^) and towards the HT for 4-NO_2_−4′-MeO-**5** (∆*G* = 2.39 kcal mol^−1^).

**Table 5:** Halogen bond strengths in the AT (∆*E*_XB–AT_) and HT (∆*E*_XB–HT_) dimers, and the ∆*G*, ∆*G*_dimer_, and ∆∆*G* values for the derivatives of **5**. All values are in kcal mol^−1^

Compound	∆*E*_XB–AT_	∆*E*_XB–HT_	∆*G*	∆*G*_dimer_	∆∆*G*
**5**	−2.91	−3.85	0.25	−1.00	−1.25
3-MeO-**5**	−4.10	−4.70	−0.86	−2.44	−1.58
3-NO_2_-**5**	−3.94	−4.63	−0.47	−1.88	−1.41
4-MeO-**5**	−2.95	−3.97	−1.52	−4.61	−3.09
4-NO_2_-**5**	−3.15	−3.97	1.13	1.00	−0.13
5-MeO-**5**	−2.88	−3.90	0.65	−0.36	−1.01
5-NO_2_-**5**	−3.00	−4.01	1.05	0.38	−0.67
6-MeO-**5**	−2.94	−3.78	0.38	−0.18	−0.56
6-NO_2_-**5**	−2.86	−3.94	0.72	−0.29	−1.01
7-MeO-**5**	−2.94	−3.97	0.61	−0.18	−0.79
7-NO_2_-**5**	−2.84	−3.83	0.70	−0.10	−0.80
8-MeO-**5**	−2.83	−3.72	−1.04	−3.16	−2.12
8-NO_2_-**5**	−2.80	−3.74	0.13	−1.42	−1.55
3′-MeO-**5**	−2.80	−3.66	−0.09	−1.32	−1.23
3′-NO_2_-**5**	−4.22	−5.48	0.01	−1.53	−1.54
4′-MeO-**5**	−2.82	−3.69	1.33	1.41	0.08
4′-NO_2_-**5**	−3.27	−4.39	−0.96	−3.43	−2.47
5′-MeO-**5**	−3.04	−4.11	−0.09	−1.00	−0.91
5′-NO_2_-**5**	−3.30	−4.35	−0.21	−1.74	−1.53
6′-MeO-**5**	−3.02	−4.11	0.86	−1.54	−2.40
6′-NO_2_-**5**	−3.32	−4.47	0.01	−1.50	−1.51
4-MeO-4′-NO_2_-**5**	−2.91	−3.85	−2.46	−6.14	−3.68
4-NO_2_−4′-MeO-**5**	−4.10	−4.70	2.39	3.86	1.11

The strength of a single halogen bond in the AT and HT dimers is denoted by ∆*E*_XB–AT_ and ∆*E*_XB–HT_, respectively. Overall, the HT dimers are more strongly bound (∆*E*_XB–HT_ = −5.48 – −3.66 kcal mol^−1^) than the AT dimers (∆*E*_XB–AT_ = −4.22 – −2.80 kcal mol^−1^), as shown in Table 5. Upon dimerisation, the equilibrium is shifted towards the HT, except for 4′-MeO-**5** (∆∆*G* = 0.08 kcal mol^−1^). Interestingly, although 4-MeO-**5** and 4-NO_2_-**5** have similar XB strengths, their behaviour upon dimerisation differs. For 4-MeO-**5**, dimerisation shifts the equilibrium noticeably towards HT (∆∆*G* = −3.09 kcal mol^−1^), whereas for 4-NO_2_-**5**, essentially no shift is observed (∆∆*G* = −0.13 kcal mol^−1^). The opposite behaviour is observed for 4′-substituted derivatives: dimerisation has little effect for 4′-MeO-**5**, but it shifts the equilibrium of 4′-NO_2_-**5** towards HT (∆∆*G* = −2.47 kcal mol^−1^). When employing two substituents, even larger shifts in ∆∆*G* are observed. The largest shift towards HT occurs for 4-MeO-4′-NO_2_-**5** (∆∆*G* = −3.68 kcal mol^−1^), whereas dimerisation shifts the equilibrium towards AT in 4-NO_2_−4′-MeO-**5** (∆∆*G* = 1.11 kcal mol^−1^).

**Fig. 7:**
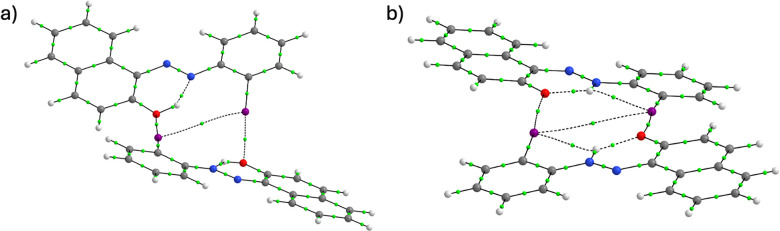
**a** The dimer of the azo tautomer of 2′-iodo-2-hydroxyazonaphthalene (AT-**5**). **b** The dimer of the hydrazone tautomer of 2′-iodo-2-hydroxyazonaphthalene (HT-**5**). The bond critical points are shown in green

The geometry of the dimers depends mainly on the orientation of the halogen bonds, as the monomers themselves are rigid and planar. AT-**5** forms a non-planar dimer due to the sp^3^-hybridised hydroxyl oxygens (Fig. 7a), whereas the HT-**5** dimer is nearly planar due to the sp^2^-hybridised keto oxygen (Fig. 7b). Although the halogen bond lengths, angles, and dihedral angles vary, all derivatives of **5** show similar dimer formation (see Table S12). The *E*_IQA_ values for the XB interaction follow the same trend as the *E*_XB_ values: the interaction is weaker in the azo tautomers (*E*_IQA_ = −63.2 − −33.5 kcal mol^−1^) than in the hydrazone tautomers (*E*_IQA_ = −70.4 − −41.9 kcal mol^−1^). As the (N)H⋅⋅⋅I distance is shorter in HT-**5** (2.725 Å) than the (O)H⋅⋅⋅I distance in AT-**5** (2.935 Å), an intramolecular iodine–hydrogen bond path is present in the HT-**5** dimer but not in the AT-**5** dimer. The *E*_IQA_ value (+ 9.6 kcal mol^−1^) indicates that this interaction is slightly destabilising.

The 3-MeO substituent increases the XB strength in both tautomers due to bifurcated halogen bonds to OH/O and MeO. Stronger halogen bonds are also observed for 3-NO_2_-**5**, which forms bifurcated halogen bonds to OH/O and a nitro oxygen. However, as the XB strength increases in both tautomers, ∆∆*G* is not significantly affected. Substituents in positions 5–8 have little effect on XB strength but slightly influence how dimerisation affects the tautomeric equilibrium. In addition to the strong I⋅⋅⋅O interaction, a weak interaction between iodine atoms is present in dimers. The *E*_IQA_ values for the iodine–iodine interaction range from −2.09 to 2.05 kcal mol^−1^ for the AT dimers, indicating that the interaction may be slightly stabilising or destabilising depending on the substituents. For all HT dimers, the iodine–iodine interaction is destabilising (*E*_IQA_ = 1.97 − 4.17 kcal mol^−1^), except for 3-NO_2_-**5**, for which it is slightly stabilising (*E*_IQA_ = −0.13 kcal mol^−1^). The bond paths are considered to indicate exchange channels which provide stabilisation due to the stabilising exchange–correlation energy (*E*_XC_ < 0), although the interaction overall may be destabilising (*E*_IQA_ > 0) [51]. By separating the iodine–iodine interactions into Coulombic (*E*_cl_) and exchange–correlation (*E*_XC_) contributions, it is seen that whereas the exchange–correlation contribution is indeed always stabilising (*E*_XC_ = −3.91 − −0.74 kcal mol^−1^), the Coulombic contribution is in all cases destabilising (*E*_cl_ = 1.42 − 4.17 kcal mol^−1^). The exact nature of the iodine–iodine interaction therefore depends on the magnitudes of the Coulombic and the exchange–correlation energy components.

Overall, the ∆*G* value for **5** is only slightly positive and can be fine-tuned by methoxy and nitro substituents. Upon dimerisation, it decreases by up to 3.68 kcal mol^−1^, suggesting that systems can be designed in which dimerisation induces inversion of the tautomeric equilibrium.

## Conclusions

We have computationally studied the azo–hydrazone tautomerisation of methoxy-substituted 2-hydroxy- and 4-hydroxyazobenzenes, and their azonaphthalene analogues. We also studied how halogen bonding by molecular iodine affects the tautomeric equilibrium. The keto group in the hydrazone tautomers forms stronger halogen bonds than the hydroxyl group in the azo tautomers. Therefore, halogen bonding generally shifts the tautomeric equilibrium towards the hydrazone tautomer. For many derivatives, the azo tautomer was only slightly favoured, and halogen bonding inverted the tautomeric equilibrium, favouring the hydrazone tautomer. External stimuli that weaken halogen bonding (e.g. temperature or competing solvents) may cancel the effect and shift the equilibrium back towards the azo tautomer. Such responsiveness could enable halogen bond-based control of azobenzene tautomerisation in sensing or thermochromic systems.

We also studied self-complementary halogen-bonded dimers based on 2′-iodo-2-hydroxyazonaphthalenes. In addition to methoxy substituents, electron-withdrawing nitro substituents were also introduced to strengthen halogen bonding by decreasing electron density on the donor atom. In general, dimerisation shifted the tautomeric equilibrium towards the hydrazone tautomer and for many derivatives inverted the tautomeric equilibrium. Such dimers may offer a tunable platform for utilising halogen bond-driven tautomerisation in sensing applications.

## Supplementary information

Below is the link to the electronic supplementary material.

ESM 1 (PDF 255 KB)

## Data Availability

All data are presented in the manuscript and the Supporting Information.
