# Supplementary material for: Halogen bond-driven azo–hydrazone tautomerisation: a computational study
Source: J Mol Model. 2026 May 7;32(6):166. doi: 10.1007/s00894-026-06740-5 (PMC13152965; doi:10.1007/s00894-026-06740-5)
Supplement: Supplementary file 1 — (PDF 255 KB) [file 894_2026_6740_MOESM1_ESM.pdf]

Supplementary information

Halogen bond-driven azobenzene tautomerisation: a computational study

Antti Siiskonen and Arri Priimagi

Smart Photonic Materials, Faculty of Engineering and Natural Sciences, Tampere University, Korkeakoulunkatu 3, FI-33720 Tampere, Finland

arri.priimagi@tuni.fi

TUNI Luottamuksellinen - Confidential (3Y)

Halogen bonding in the derivatives of 1 – 4.

The acceptor sites for **1** are presented in **Fig. S1**. Same nomenclature is used for **2** – **4**. The propensity of OH/O, N1, and N2 to act as XB acceptors varies across the core structures. Whereas OH/O and N1 act as halogen bond acceptors in all compounds for both tautomers, N2 does so only in the derivatives of **1**, AT-**2** and **3**. The N2 atom is sp^3-hydridised in HTs but due to conjugation it is nevertheless planar. To act as an XB acceptor, it needs to lose planarity which weakens the internal hydrogen bond in **2** and **4** by increasing its length and consequently N2 is a poor acceptor site in HT-**2** and HT-**4**.

Hybridisation of the XB acceptor atom (sp^2 or sp^3) dictates the orientation of the halogen bond as the XB donor preferably approaches from the direction of the free electron pair on the acceptor. N1 is sp^2-hybridised in both tautomers, and the halogen-bonded complex would be expected to be planar. However, steric hindrance prevents in-plane approach. Same is observed for N2 in the ATs. Therefore, binding to N1 or N2 requires azobenzene or azonaphthalene to adopt a non-planar geometry to allow better access to the acceptor site. That increases the energy of the complexes and diminishes the energy-lowering effect of XB when the acceptor site is N1 or N2. Binding to OH/O does not require similar changes in geometry. Therefore, although iodine may interact more strongly with N1 or N2 than with OH/O, the lowest-energy complex may nevertheless have XB to OH/O.

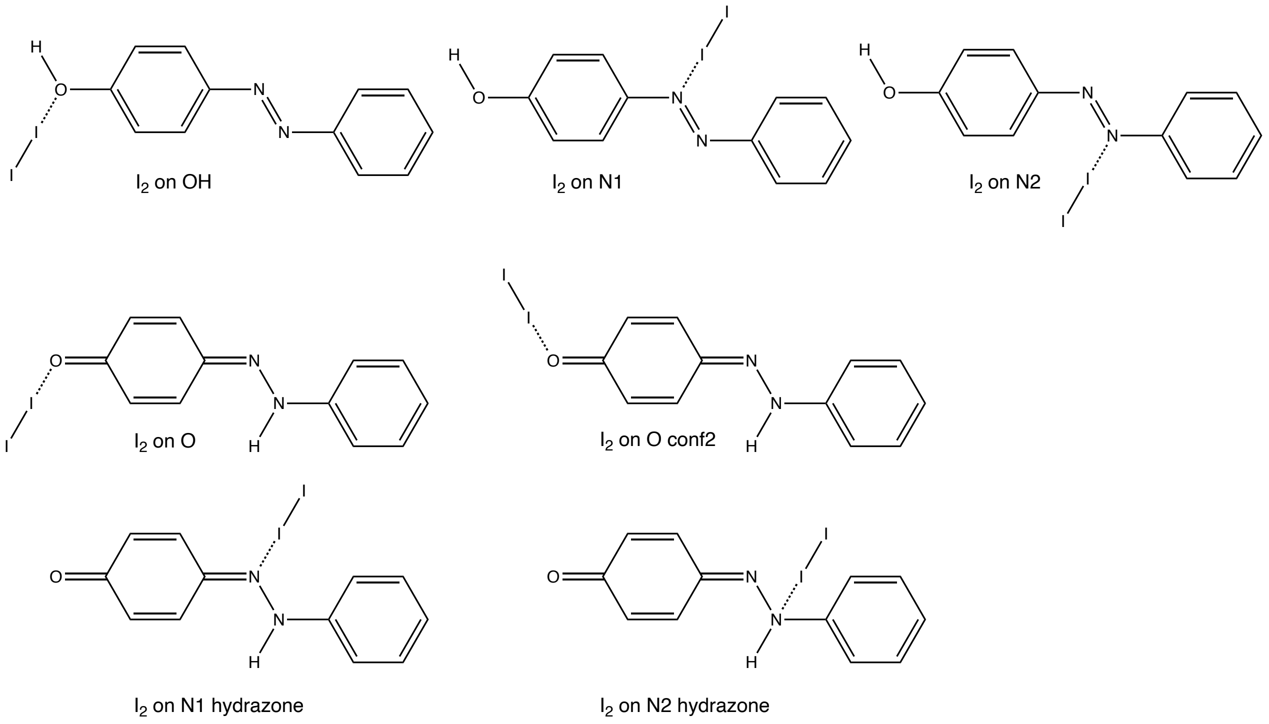

Fig. S1 The XB acceptor sites in AT-**1** and HT-**1**.

Table S1 The Δ$G_{XB–AT}$ and Δ$G_{XB–HT}$ values for the derivatives of **1**. The column label (e.g. on OH) denotes the site of halogen bonding. All values are in kcal/mol.

Compound	Δ$G_{XB–AT}$			Δ$G_{XB–HT}$			
	on OH	on N1	on N2	on O	on O conf2	on N1	on N2
**1**	0	0.12	0.17	7.29	7.33	9.64	10.00
2-MeO-**1**	1.06	0	0.98	0.54	1.12	3.73	3.78
3-MeO-**1**	0.15	0.49	0	9.85	8.45	11.63	11.96
2'-MeO-**1**	0.90	0.83	0	4.36	4.55	6.83	6.84
3'-MeO-**1**	0.47	0	0.53	7.29	7.09	9.54	10.11
4'-MeO-**1**	0.05	0.11	0	8.68	8.52	11.19	11.69
2-MeO-2'-MeO-**1**	4.04	3.24	2.64	0	0.29	3.33	2.33

Table S2 XB strengths for the complexes of iodine and the derivatives of **1**. The column label (e.g. on OH) denotes the site of halogen bonding. The value for the complex with the lowest Δ*G* value for the given tautomer is shown in bold. All values are in kcal/mol.

Compound	$E_{XB–AT}$			$E_{XB–HT}$			
	on OH	on N1	on N2	on O	on O conf2	on N1	on N2
**1**	**-4.33**	-6.57	-6.36	**-7.28**	-7.21	-4.73	-4.45
2-MeO-**1**	-4.33	**-8.21**	-7.23	**-8.56**	-7.93	-4.63	-4.66
3-MeO-**1**	-4.68	-7.03	**-7.09**	-6.89	**-7.96**	-5.70	-5.96
2'-MeO-**1**	-4.47	-7.27	**-8.10**	**-7.60**	-7.51	-4.89	-5.23
3'-MeO-**1**	-4.32	**-6.98**	-6.30	-7.27	**-7.24**	-5.10	-4.44
4'-MeO-**1**	-4.45	-6.61	**-6.64**	-7.61	**-7.52**	-4.96	-4.68
2-MeO-2'-MeO-**1**	-4.74	**-8.92**	-8.98	**-8.90**	-8.25	-4.75	-5.76

Table S3. The Δ$G_{XB–AT}$ and Δ$G_{XB–HT}$ values for the derivatives of **2**. The column label (e.g. on OH) denotes the site of halogen bonding. All values are in kcal/mol.

Compound	Δ$G_{XB–AT}$			Δ$G_{XB–HT}$	
	on OH	on N1	on N2	on O	on N1
**2**	0	1.36	1.57	4.21	7.37
3-MeO-**2**	0	2.97	3.20	3.85	7.49
4-MeO-**2**	0	1.36	1.53	1.16	4.51
5-MeO-**2**	0	0.76	1.37	2.75	5.38
6-MeO-**2**	0.88	0	2.30	2.98	4.71
2'-MeO-**2**	0	1.87	1.54	3.07	7.22
3'-MeO-**2**	0	0.93	1.30	4.04	6.81
4'-MeO-**2**	0	1.26	1.39	4.81	8.16
4-MeO-6-MeO-**2**	0.59	0	2.10	0.12	2.17

Table S4. XB strengths for the complexes of iodine and the derivatives of **2**. The column label (e.g. on OH) denotes the site of halogen bonding. The value for the complex with the lowest ΔG value for the given tautomer is shown in bold. All values are in kcal/mol.

Compound	E_{XB-AT}			E_{XB-HT}	
	on OH	on N1	on N2	on O	on N1
**2**	**-5.09**	-5.50	-3.60	**-7.67**	-4.51
3-MeO-**2**	**-7.04**	-5.59	-3.64	**-8.59**	-4.66
4-MeO-**2**	**-5.34**	-5.72	-3.95	**-8.40**	-4.69
5-MeO-**2**	**-5.44**	-5.40	-3.74	**-7.82**	-4.68
6-MeO-**2**	-5.22	**-7.12**	-3.88	**-7.95**	-6.46
2'-MeO-**2**	**-6.09**	-5.85	-5.60	**-8.84**	-4.68
3'-MeO-**2**	**-5.07**	-5.88	-3.65	**-7.66**	-4.94
4'-MeO-**2**	**-5.30**	-5.55	-3.79	**-8.17**	-4.67
4-MeO-6-MeO-**2**	**-5.50**	-7.27	-4.22	**-8.69**	-6.47

Table S5. The ΔG_{XB-AT} and ΔG_{XB-HT} values for the derivatives of **3**. The column label (e.g. on OH) denotes the site of halogen bonding. All values are in kcal/mol.

Compound	ΔG_{XB-AT}			ΔG_{XB-HT}			
	on OH	on N1	on N2	on O	on O conf2	on N1	on N2
**3**	1.27	1.43	0.72	0	0.68	1.73	2.67
2-MeO-**3**	12.57	11.85	10.05	0	0.69	2.60	2.91
3-MeO-**3**	1.25	1.82	0.81	0	1.13	3.12	3.07
5-MeO-**3**	0	1.13	0.74	6.44	5.95	8.89	9.48
6-MeO-**3**	5.62	6.46	5.60	0	5.26	6.92	7.74
7-MeO-**3**	1.18	0.79	0.35	0	0.37	1.56	2.60
8-MeO-**3**	3.33	1.02	1.04	1.02	0	1.65	3.86
2'-MeO-**3**	5.11	4.84	3.08	0	2.58	1.53	1.88
3'-MeO-**3**	1.33	1.17	0.76	0	0.46	1.50	2.61
4'-MeO-**3**	0.32	0.73	0	1.29	1.60	2.82	3.81

Table S6. XB strengths for the complexes of iodine and the derivatives of **3**. The column label (e.g. on OH) denotes the site of halogen bonding. The value for the complex with the lowest ΔG value for the given tautomer is shown in bold. All values are in kcal/mol.

Compound	E_{XB-AT}			E_{XB-HT}			
	on OH	on N1	on N2	on O	on O conf2	on N1	on N2
**3**	-4.29	-6.19	**-6.92**	**-6.86**	-6.02	-5.12	-5.31
2-MeO-**3**	-4.30	-7.42	**-9.97**	**-8.16**	-6.62	-4.97	-5.47
3-MeO-**3**	-4.65	-5.95	**-7.40**	**-7.61**	-5.76	-6.11	-6.88
5-MeO-**3**	**-5.02**	-6.32	-6.84	-7.38	**-8.38**	-5.55	-5.61
6-MeO-**3**	-4.90	-6.20	**-6.97**	**-6.91**	-6.91	-5.23	-5.56
7-MeO-**3**	-4.31	-6.77	**-6.93**	**-7.11**	-6.23	-5.72	-5.36
8-MeO-**3**	-4.39	**-9.31**	-8.48	-7.06	**-6.12**	-8.16	-5.64
2'-MeO-**3**	-4.41	-6.83	**-8.64**	**-7.10**	-6.21	-5.26	-6.08
3'-MeO-**3**	-4.28	-6.61	**-6.88**	**-6.83**	-6.01	-5.35	-5.36
4'-MeO-**3**	-4.39	-6.28	**-7.22**	**-7.10**	-6.19	-5.32	-5.67

Table S7. The $\Delta G_{XB\text{–}AT}$ and $\Delta G_{XB\text{–}HT}$ values for the derivatives of **4**. The column label (e.g. on OH) denotes the site of halogen bonding. All values are in kcal/mol.

Compound	$\Delta G_{XB\text{–}AT}$		$\Delta G_{XB\text{–}HT}$	
	on OH	on N1	on O	on N1
**4**	1.81	2.87	0	2.39
3-MeO-**4**	1.75	4.44	0	2.87
4-MeO-**4**	3.82	4.92	0	2.86
5-MeO-**4**	1.23	2.54	0	2.24
6-MeO-**4**	1.04	2.30	0	2.13
7-MeO-**4**	1.21	2.23	0	2.32
8-MeO-**4**	2.77	2.80	0	1.41
2'-MeO-**4**	1.71	3.92	0	2.89
3'-MeO-**4**	1.11	2.31	0	1.86
4'-MeO-**4**	0.49	1.52	0	2.36

Table S8. XB strengths for the complexes of iodine and the derivatives of **4**. The column label (e.g. on OH) denotes the site of halogen bonding. The value for the complex with the lowest ΔG value for the given tautomer is shown in bold. All values are in kcal/mol.

Name	$E_{XB\text{–}AT}$		$E_{XB\text{–}HT}$	
	on OH	on N1	on O	on N1
**4**	**-4.86**	-5.12	**-6.95**	-4.69
3-MeO-**4**	**-6.79**	-5.13	**-8.04**	-4.71
4-MeO-**4**	**-5.28**	-5.33	**-7.85**	-4.87
5-MeO-**4**	**-4.95**	-5.23	**-7.20**	-4.78
6-MeO-**4**	**-5.03**	-5.14	**-7.06**	-4.79
7-MeO-**4**	**-4.96**	-5.75	**-7.13**	-5.22
8-MeO-**4**	**-5.05**	-8.15	**-7.15**	-7.33
2'-MeO-**4**	**-5.90**	-5.53	**-7.92**	-4.88
3'-MeO-**4**	**-4.85**	-5.47	**-6.92**	-5.06
4'-MeO-**4**	**-5.08**	-5.25	**-7.33**	-4.80

Table S9. IQA interatomic energies (E_{IQA}), and the coulombic (E_{cl}) and exchange-correlation (E_{XC}) contributions of the iodine-oxygen interaction in AT-**4** and HT-**4** derivatives. All values are in Hartrees.

Name	**azo tautomer** iodine-oxygen interaction			**hydrazone tautomer** iodine-oxygen interaction		
	E_{IQA}	E_{Cl}	E_{XC}	E_{IQA}	E_{Cl}	E_{XC}
**4**	-0.0642	-0.0398	-0.0244	-0.0860	-0.0530	-0.0330
3-MeO-**4**	-0.1035	-0.0719	-0.0316	-0.1202	-0.0828	-0.0374
4-MeO-**4**	-0.0654	-0.0409	-0.0246	-0.0938	-0.0581	-0.0357
5-MeO-**4**	-0.0654	-0.0408	-0.0246	-0.0887	-0.0549	-0.0338
6-MeO-**4**	-0.0660	-0.0410	-0.0250	-0.0871	-0.0537	-0.0333
7-MeO-**4**	-0.0652	-0.0405	-0.0246	-0.0880	-0.0544	-0.0336
8-MeO-**4**	-0.0686	-0.0401	-0.0286	-0.0881	-0.0544	-0.0337
2'-MeO-**4**	-0.0726	-0.0459	-0.0267	-0.0933	-0.0581	-0.0352
3'-MeO-**4**	-0.0642	-0.0398	-0.0244	-0.0858	-0.0529	-0.0329

4'-MeO-**4**	-0.0665	-0.0413	-0.0252	-0.0900	-0.0556	-0.0344

Table S10. IQA interatomic energies (E_{IQA}), and the coulombic (E_{cl}) and exchange-correlation (E_{XC}) contributions of the iodine-oxygen interaction in the dimers of the AT-**5** and HT-**5** derivatives. All values are in Hartrees.

Name	**azo tautomer** iodine-oxygen interaction			**hydrazone tautomer** iodine-oxygen interaction		
	E_{IQA}	E_{Cl}	E_{XC}	E_{IQA}	E_{Cl}	E_{XC}
**5**	-0.0577	-0.0402	-0.0174	-0.0693	-0.0483	-0.0211
3-MeO-**5**	-0.1024	-0.0776	-0.0248	-0.1121	-0.0826	-0.0295
3-NO_2-**5**	-0.0746	-0.0484	-0.0263	-0.0881	-0.0585	-0.0296
4-MeO-**5**	-0.0551	-0.0379	-0.0173	-0.0703	-0.0486	-0.0217
4-NO_2-**5**	-0.0577	-0.0404	-0.0172	-0.0683	-0.0477	-0.0205
5-MeO-**5**	-0.0576	-0.0401	-0.0175	-0.0707	-0.0493	-0.0214
5-NO_2-**5**	-0.0568	-0.0396	-0.0172	-0.0698	-0.0489	-0.0209
6-MeO-**5**	-0.0581	-0.0404	-0.0177	-0.0687	-0.0477	-0.0210
6-NO_2-**5**	-0.0557	-0.0387	-0.0170	-0.0696	-0.0487	-0.0209
7-MeO-**5**	-0.0579	-0.0404	-0.0175	-0.0709	-0.0496	-0.0214
7-NO_2-**5**	-0.0555	-0.0385	-0.0170	-0.0681	-0.0474	-0.0207
8-MeO-**5**	-0.0563	-0.0390	-0.0173	-0.0679	-0.0470	-0.0209
8-NO_2-**5**	-0.0533	-0.0366	-0.0167	-0.0672	-0.0466	-0.0205
3'-MeO-**5**	-0.0628	-0.0400	-0.0228	-0.0728	-0.0499	-0.0229
3'-NO_2-**5**	-0.0755	-0.0501	-0.0254	-0.0880	-0.0581	-0.0300
4'-MeO-**5**	-0.0603	-0.0387	-0.0216	-0.0725	-0.0474	-0.0251
4'-NO_2-**5**	-0.0634	-0.0423	-0.0211	-0.0735	-0.0494	-0.0241
5'-MeO-**5**	-0.0567	-0.0363	-0.0204	-0.0668	-0.0435	-0.0233
5'-NO_2-**5**	-0.0648	-0.0434	-0.0214	-0.0757	-0.0511	-0.0246
6'-MeO-**5**	-0.0590	-0.0364	-0.0227	-0.0730	-0.0480	-0.0250
4-MeO-4'-NO_2-**5**	-0.0617	-0.0441	-0.0176	-0.0776	-0.0552	-0.0224
4-NO_2-4'-MeO-**5**	-0.0604	-0.0427	-0.0178	-0.0728	-0.0512	-0.0215

Table S11. IQA interatomic energies (E_{IQA}), and the coulombic (E_{cl}) and exchange-correlation (E_{XC}) contributions of the iodine-iodine interaction interaction in the dimers of the AT-**5** and HT-**5** derivatives. All values are in Hartrees.

Name	**azo tautomer** iodine-iodine interaction			**hydrazone tautomer** iodine-iodine interaction		
	E_{IQA}	E_{Cl}	E_{XC}	E_{IQA}	E_{Cl}	E_{XC}
**5**	-0.0007	0.0026	-0.0033	0.0006	0.0033	-0.0027
3-MeO-**5**	0.0017	0.0029	-0.0012	0.0014	0.0035	-0.0021
3-NO_2-**5**	-0.0016	0.0031	-0.0047	-0.0002	0.0039	-0.0041
4-MeO-**5**	-0.0006	0.0023	-0.0029	0.0006	0.0033	-0.0026
4-NO_2-**5**	-0.0003	0.0027	-0.0029	0.0007	0.0034	-0.0027
5-MeO-**5**	0.0006	0.0026	-0.0020	0.0007	0.0035	-0.0027
5-NO_2-**5**	-0.0008	0.0026	-0.0035	0.0006	0.0035	-0.0028
6-MeO-**5**	-0.0007	0.0027	-0.0033	0.0005	0.0033	-0.0028
6-NO_2-**5**	-0.0010	0.0025	-0.0035	0.0005	0.0035	-0.0030
7-MeO-**5**	-0.0007	0.0027	-0.0034	0.0007	0.0035	-0.0028

7-NO_2-**5**	-0.0008	0.0024	-0.0032	0.0006	0.0033	-0.0027
8-MeO-**5**	-0.0012	0.0025	-0.0037	0.0004	0.0032	-0.0028
8-NO_2-**5**	-0.0018	0.0023	-0.0041	0.0005	0.0031	-0.0027
3'-MeO-**5**	0.0010	0.0033	-0.0023	0.0020	0.0041	-0.0021
3'-NO_2-**5**	0.0033	0.0056	-0.0023	0.0043	0.0066	-0.0024
4'-MeO-**5**	-0.0008	0.0029	-0.0037	0.0006	0.0037	-0.0031
4'-NO_2-**5**	0.0004	0.0036	-0.0033	0.0017	0.0043	-0.0026
5'-MeO-**5**	-0.0006	0.0025	-0.0031	0.0006	0.0032	-0.0026
5'-NO_2-**5**	0.0005	0.0038	-0.0033	0.0020	0.0046	-0.0026
6'-MeO-**5**	-0.0033	0.0029	-0.0062	0.0007	0.0035	-0.0028
6'-NO_2-**5**	-0.0020	0.0038	-0.0057	0.0018	0.0045	-0.0027
4-MeO-4'-NO_2-**5**	0.0009	0.0033	-0.0024	0.0019	0.0044	-0.0025
4-NO_2-4-MeO-**5**	0.0001	0.0030	-0.0028	0.0010	0.0038	-0.0028

Table S12. The halogen bond lengths (top), angles (middle) and dihedral angles (bottom) in the complexes of iodine with the derivatives of **1**. The column label (e.g. on OH) denotes the site of halogen bonding on the acceptor. The lengths are in Ångströms and the angles are in degrees.

Name	**azo tautomer**			**hydrazone tautomer**			
	on OH	on N1	on N2	on O	on O conf2	on N1	on N2
**1**	3.003	3.045	3.035	2.826	2.826	3.147	3.113
	178.53	174.17	174.34	179.02	179.67	174.13	172.71
	47.99	43.54	50.03	0.01	0.03	55.00	82.06
2-MeO-**1**	3.006	3.072	2.989	2.773	2.792	3.165	3.183
	178.25	168.74	174.92	178.88	179.93	174.80	175.20
	48.90	51.83	46.98	0.03	0.04	58.95	79.59
3-MeO-**1**	2.986	3.052	3.036	2.846	2.846	3.111	3.044
	178.12	174.10	175.97	178.67	179.31	174.17	174.55
	49.50	44.12	50.98	0.01	0.02	50.06	82.60
2'-MeO-**1**	2.994	3.002	3.063	2.810	2.811	3.139	3.119
	178.76	174.48	168.20	179.43	179.73	174.68	175.35
	46.80	41.32	54.47	0.00	0.00	56.03	71.97
3'-MeO-**1**	3.005	3.056	3.045	2.825	2.825	3.167	3.123
	178.27	174.62	174.41	179.24	179.61	175.10	172.33
	50.04	45.05	49.23	0.00	0.02	53.12	85.32
4'-MeO-**1**	2.997	3.030	3.031	2.809	2.810	3.130	3.091
	178.47	174.38	174.48	179.38	179.92	174.31	173.30
	49.52	47.54	46.90	0.01	0.02	52.81	81.59
2-MeO-4'-MeO-**1**	2.997	3.060	2.983	2.758	2.778	3.150	3.167
	178.19	168.68	175.07	179.06	179.74	174.80	175.61
	49.08	53.46	43.20	0.07	0.08	57.41	78.80

Table S13. The halogen bond lengths (top), angles (middle) and dihedral angles (bottom) in the complexes of iodine with the derivatives of **2**. The column label (e.g. on OH) denotes the site of halogen bonding on the acceptor. The lengths are in Ångströms and the angles are in degrees.

Name	azo tautomer			hydrazone tautomer	
	on OH	on N1	on N2	on O	on N1
**2**	2.957	3.111	3.249	2.801	3.162
	179.08	172.57	176.55	177.98	175.14
	41.73	45.44	69.69	0.02	53.19
3-MeO-**2**	3.094	3.107	3.248	2.895	3.152
	162.50	173.72	177.53	169.24	174.77
	0.05	45.82	69.29	0.07	54.12
4-MeO-**2**	2.958	3.098	3.226	2.774	3.152
	178.91	173.60	176.97	178.80	175.23
	39.17	45.12	70.62	0.01	53.59
5-MeO-**2**	2.946	3.108	3.241	2.816	3.139
	179.18	174.17	177.38	178.31	175.65
	45.36	43.14	68.54	0.01	51.66
6-MeO-**2**	2.952	3.271	3.228	2.788	3.389
	179.20	155.66	177.08	177.95	148.45
	39.11	46.03	71.26	0.00	47.05
2'-MeO-**2**	2.910	3.073	3.499	2.756	3.153
	178.71	174.37	144.82	177.45	175.66
	36.74	48.05	52.37	0.02	54.63
3'-MeO-**2**	2.959	3.131	3.251	2.801	3.181
	178.88	174.50	174.71	178.22	176.48
	43.10	45.66	71.36	0.01	51.58
4'-MeO-**2**	2.944	3.098	3.238	2.779	3.146
	179.07	174.19	177.60	177.86	175.13
	42.62	48.77	69.80	0.06	52.59
2-MeO-4'-MeO-**2**	2.997	3.060	2.983	2.758	3.150
	178.19	168.68	175.07	179.06	174.80
	49.08	53.46	43.75	0.07	57.41

Table S14. The halogen bond lengths (top), angles (middle) and dihedral angles (bottom) in the complexes of iodine with the derivatives of **3**. The column label (e.g. on OH) denotes the site of halogen bonding on the acceptor. The lengths are in Ångströms and the angles are in degrees.

Name	azo tautomer			hydrazone tautomer			
	on OH	on N1	on N2	on O	on O conf2	on N1	on N2
**3**	3.050	3.066	3.022	2.845	2.881	3.167	3.044
	174.75	174.05	174.71	179.44	179.84	173.11	175.50
	72.06	66.43	42.22	0.40	0.02	66.86	86.04
2-MeO-**3**	3.057	2.984	2.946	2.793	2.851	3.189	3.124
	173.81	174.17	173.55	178.93	179.80	172.33	176.48
	73.60	71.47	32.34	0.24	0.02	70.53	82.70
3-MeO-**3**	3.029	3.093	3.016	2.940	2.895	3.115	2.981

	175.57	174.37	176.17	171.81	178.96	172.87	177.42
	73.04	65.60	47.13	0.35	0.01	62.66	82.68
5-MeO-**3**	2.962	3.070	3.013	2.821	2.868	3.149	3.025
	178.72	174.44	174.42	179.78	172.80	172.54	176.35
	49.67	65.95	44.30	0.04	48.35	64.03	85.63
6-MeO-**3**	3.042	3.064	3.022	2.841	2.881	3.157	3.027
	174.47	174.46	174.50	179.68	178.40	173.52	175.67
	67.69	66.27	42.50	0.31	0.00	66.98	85.60
7-MeO-**3**	3.048	3.080	3.020	2.833	2.868	3.169	3.041
	175.14	174.76	174.50	179.46	179.49	173.24	175.92
	72.35	63.68	41.77	0.02	16.08	63.84	87.97
8-MeO-**3**	3.044	2.984	2.952	2.835	2.874	3.145	3.034
	173.98	172.73	174.79	179.53	179.22	164.33	175.77
	73.30	59.89	36.73	1.58	22.75	51.70	81.42
2'-MeO-**3**	3.043	3.026	3.031	2.834	2.871	3.159	3.041
	174.78	174.10	169.70	179.47	179.91	173.53	176.89
	72.32	64.49	52.85	0.27	0.03	67.45	74.14
3'-MeO-**3**	3.050	3.079	3.030	2.847	2.881	3.183	3.050
	174.60	174.77	174.93	179.44	179.47	174.56	175.59
	72.26	67.01	40.30	0.36	15.90	66.08	90.25
4'-MeO-**3**	3.042	3.056	3.016	2.834	2.872	3.161	3.015
	174.49	174.48	174.68	179.44	179.93	173.61	176.15
	72.52	68.18	39.01	0.27	0.08	65.66	88.79

Table S15. The halogen bond lengths (top), angles (middle) and dihedral angles (bottom) in the complexes of iodine with the derivatives of **4**. The column label (e.g. on OH) denotes the site of halogen bonding on the acceptor. The lengths are in Ångströms and the angles are in degrees.

Name	**azo tautomer**		**hydrazone tautomer**	
	on OH	on N1	on O	on N1
**4**	2.968	3.164	2.835	3.193
	179.34	173.63	178.25	173.41
	37.87	64.22	0.08	68.04
3-MeO-**4**	3.102	3.165	2.932	3.195
	162.31	173.12	168.52	172.86
	0.02	64.22	0.19	68.14
4-MeO-**4**	2.964	3.155	2.801	3.183
	178.81	174.75	178.99	174.53
	37.33	64.28	0.04	68.36
5-MeO-**4**	2.96	3.16	2.82	3.20
	179.39	173.07	178.07	171.26
	35.41	64.19	0.07	68.07
6-MeO-**4**	2.958	3.161	2.830	3.183
	179.46	174.15	178.46	174.68
	37.53	64.27	0.01	68.10
7-MeO-**4**	2.963	3.171	2.826	3.198
	179.35	174.75	178.16	174.21
	37.30	62.32	0.09	65.57
8-MeO-**4**	2.954	3.131	2.826	3.28

	179.13	166.18	178.14	154.50
	7.39	54.21	1.64	62.68
2'-MeO-**4**	2.917	3.127	2.796	3.184
	178.05	174.25	177.77	173.98
	27.86	64.28	0.11	68.43
3'-MeO-**4**	2.969	3.182	2.836	3.209
	179.32	174.74	178.38	175.11
	37.94	64.19	0.06	66.53
4'-MeO-**4**	2.956	3.151	2.816	3.182
	179.10	174.13	177.98	173.54
	42.89	65.56	0.04	67.72

Fig. S2 shows how the bond lengths, angles, and dihedral angles are defined for the iodine–oxygen, iodine–iodine, and hydrogen bond interactions in the dimers of the derivatives **5**. The bond angle for the iodine-oxygen interaction refers to the C2'-I-O angle, and the bond angle for the iodine-iodine interaction refers to the C2'-I-I angle. The dihedral angles for the iodine-oxygen and iodine-iodine interactions are shown in blue and red, respectively. The bond angles for intramolecular hydrogen bonds refer to the O-H-N2 angle and the dihedral angle is defined by atoms C2, O, H and N2. In **Table S16**, the distances (top), angles (middle), and dihedral angles (bottom) are presented for all dimers of the AT-**5** and HT-**5** derivatives. For example, the atom distance for the iodine-oxygen interaction in the AT-**5** dimer is 3.129 Å, the angle is 178.14 degrees, and the dihedral angle is 36.13 degrees.

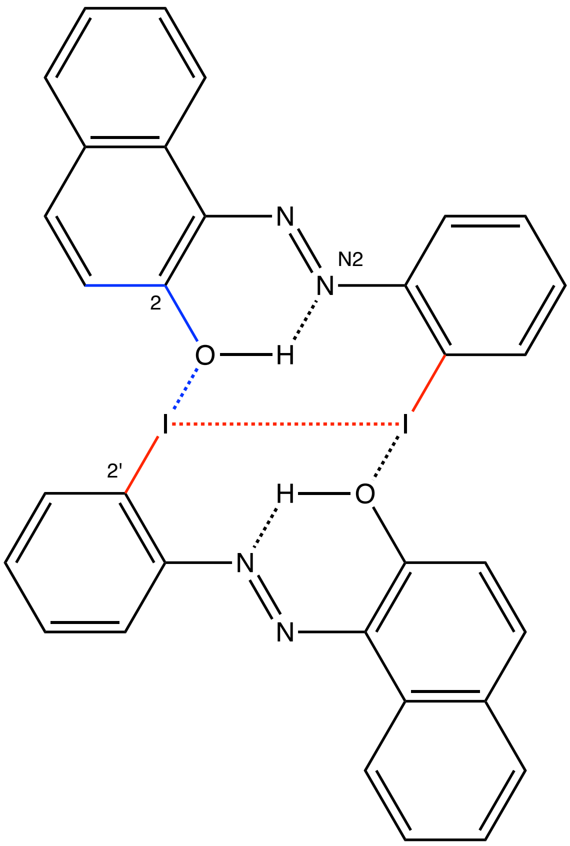

Fig. S2 The iodine–oxygen, iodine–iodine, and the hydrogen bond interactions in the dimer of AT-**5**.

Table S16. Bond distances, angles and dihedral angles for the iodine-oxygen, iodine-iodine, and hydrogen bonding interactions in the dimers of the AT-**5** and HT-**5** derivatives. All distances are in Ångströms and the angles are in degrees.

Name	**azo tautomer dimer**			**hydrazone tautomer dimer**		
	I···O	I···I	OH···N	I···O	I···I	NH···O
**5**	3.129	4.671	1.742	3.040	4.735	1.801
	178.14	128.85	139.78	176.59	128.46	130.88
	36.13	145.90	0.62	11.98	164.14	1.03
3-MeO-**5**	3.247	5.163	1.755	3.111	4.898	1.820
	166.91	121.24	139.05	172.87	121.94	129.75
	30.09	129.11	3.23	26.96	135.96	4.22
3-NO_2-**5**	3.194	4.520	1.707	3.098	4.569	1.809
	163.73	130.28	141.07	167.47	128.93	130.08
	48.52	130.76	3.66	37.14	143.82	0.85
4-MeO-**5**	3.134	4.719	1.712	3.027	4.748	1.802
	179.53	128.43	141.01	177.83	127.14	130.98
	35.32	146.87	0.40	18.48	156.86	1.29
4-NO_2-**5**	3.129	4.698	1.758	3.050	4.717	1.796
	176.25	131.46	138.69	175.80	129.54	131.03
	30.17	155.91	0.45	11.66	168.04	0.42
5-MeO-**5**	3.127	4.672	1.739	3.034	4.725	1.795

	178.02	128.88	139.91	176.57	128.37	131.07
	35.89	146.06	0.66	12.57	163.59	1.01
5-NO_2-**5**	3.135	4.647	1.751	3.040	4.706	1.795
	177.54	128.86	139.16	176.53	128.32	131.16
	40.83	143.30	0.04	16.74	159.13	1.04
6-MeO-**5**	3.123	4.667	1.753	3.041	4.721	1.802
	178.36	128.45	139.50	176.77	127.95	130.94
	37.87	144.36	0.85	16.55	158.92	1.24
6-NO_2-**5**	3.141	4.643	1.744	3.041	4.688	1.796
	178.04	128.68	139.60	176.65	127.88	131.15
	38.74	142.91	0.83	19.30	155.84	1.63
7-MeO-**5**	3.126	4.661	1.738	3.032	4.718	1.795
	178.10	128.63	140.06	176.77	128.00	131.04
	37.86	145.13	0.60	14.65	160.39	1.29
7-NO_2-**5**	3.139	4.686	1.758	3.048	4.734	1.808
	178.42	128.99	139.19	176.57	128.55	130.81
	35.05	145.44	1.02	13.82	162.16	1.14
8-MeO-**5**	3.130	4.631	1.677	3.044	4.743	1.788
	178.45	127.69	140.93	177.10	127.02	129.40
	29.56	144.81	5.14	14.18	160.84	1.06
8-NO_2-**5**	3.130	4.631	1.677	3.044	4.743	1.788
	178.46	127.70	140.93	177.10	127.02	129.40
	29.61	144.81	5.17	14.20	160.84	1.06
3'-MeO-**5**	3.125	4.797	1.75	3.045	4.827	1.807
	178.42	4.80	139.36	177.85	129.01	130.54
	20.51	130.89	0.43	0.01	179.99	0.00
3'-NO_2-**5**	3.054	4.738	1.726	2.964	4.725	1.791
	176.30	132.94	140.19	176.69	129.74	130.97
	6.50	170.86	1.03	7.66	170.10	1.46
4'-MeO-**5**	3.118	4.622	1.745	3.018	4.673	1.787
	178.64	127.19	139.92	177.10	126.60	131.45
	41.20	139.78	1.34	20.96	152.32	1.84
4'-NO_2-**5**	3.111	4.664	1.728	3.026	4.732	1.805
	177.24	129.14	140.14	176.03	129.06	130.60
	35.33	147.99	0.60	0.02	179.97	0.01
5'-MeO-**5**	3.133	4.696	1.744	3.049	4.751	1.803
	178.34	129.05	139.62	176.60	128.67	130.75
	32.86	148.48	0.65	8.64	169.08	0.63
5'-NO_2-**5**	3.105	4.667	1.734	3.015	4.740	1.804
	178.52	127.66	140.08	176.97	127.48	130.40
	37.12	143.79	1.62	14.49	161.79	1.35
6'-MeO-**5**	3.159	4.387	1.769	3.014	4.773	1.805
	173.72	127.30	139.67	179.20	123.66	131.08
	63.38	130.35	1.90	21.45	154.66	4.21
6'-NO_2-**5**	3.130	4.442	1.753	3.001	4.760	1.812
	175.28	123.98	139.51	178.76	125.06	129.72
	62.70	127.66	4.17	2.25	154.16	3.03
4-MeO-4'-NO_2-**5**	3.112	4.772	1.686	3.006	4.751	1.799
	176.82	131.81	141.82	176.81	128.63	130.93
	0.01	179.96	0.00	0.07	179.92	0.00
4-NO_2-4'-MeO-**5**	3.112	4.703	1.757	3.027	4.695	1.777

176.18	131.73	138.94	175.92	129.23	131.87
25.05	159.04	0.510	13.66	166.61	0.29
